# Predicting Growth Markers for Effective Palatal Expansion: A Comparative Study of DMPS, SMPS, and CVM 

**DOI:** 10.30476/dentjods.2025.103814.2478

**Published:** 2026-06-01

**Authors:** Farzaneh Golfeshan, Amir Hossein Hajmohammadi, Hooman Zarif Najafi, Fatemeh Dehghani

**Affiliations:** 1 Orthodontic Research Center, School of Dentistry, Shiraz University of Medical Sciences, Shiraz, Iran.; 2 Postgraduate Student, Orthodontic Research Center, School of Dentistry, Shiraz University of Medical Sciences, Shiraz, Iran.; 3 Graduated Student of Dentistry, Student Research Committee, School of Dentistry, Shiraz University of Medical Sciences, Shiraz, Iran.

**Keywords:** Sutures, Cervical vertebrae, Age, Cone-beam computed tomography (CBCT)

## Abstract

**Background:**

Effective planning and prediction of outcomes in orthodontic treatments require clinicians to identify precise growth markers. These markers play a critical role in determining the appropriate treatment strategy for patients.

**Purpose:**

The present study was conducted to evaluate the relationship between midpalatal suture density (DMPS), the stage of maturation and morphology of the midpalatal suture (SMPS), and cervical vertebra maturation (CVM) across age groups and sexes to provide insight into their interdependence.

**Materials and Method:**

The sample size of this cross-sectional study was estimated using the sample size tables for logistic regression, with α=0.05 and a power of 80%. This study analyzed archived CBCT images and lateral cephalograms of 80 patients aged 7–30 years. The sample was divided into eight age groups. Spearman’s correlation coefficient was used to assess the relationships between DMPS, SMPS, and CVM. p Value< 0.05 was considered significant.

**Results:**

The findings revealed significant correlations between CVM, DMPS, and SMPS in both males and females. Among females, the strongest correlations were between age and DMPS (r= 0.791), age and CVM (r= 0.750), and CVM and DMPS (r= 0.769). Similar trends were observed in males, with the highest correlations between age and DMPS (r= 0.832) and CVM and DMPS (r= 0.805). Across all age groups, DMPS showed the highest correlation with age, while SMPS exhibited the lowest.

**Conclusion:**

A highly significant relationship was identified between the SMPS, DMPS, and CVM parameters at all ages and in both sexes. The highest correlation belonged to age and DMPS, and the lowest correlation belonged to age and SMPS. Therefore, it can be concluded that DMPS can be estimated based on the patient's age, and vice versa to some extent. Furthermore, the correlation was stronger in men than in women, indicating a higher likelihood of a relationship.

## Introduction

In orthodontic treatments, for proper and effective treatment planning, clinicians need to determine the growth markers in patients. One such treatment is palatal expansion, which is performed in patients with narrow palatal. In addition to cervical vertebral maturation (CVM), the assessment of growth markers in these patients entails two parameters including the density of the midpalatal suture (DMPS), and the stage of maturation and morphology of the midpalatal suture (SMPS) [ 1 ].

In the past, orthodontic practices have been contingent upon two primary methodologies including the chronological age of patients or the use of CVM. The former approach entailed the augmentation of the palatal width in the context of rapid maxillary expansion (RME), a less aggressive procedure. Conversely, surgical assistance in rapid maxillary expansion (SARME) has been employed as a more pronounced intervention. In the first approach, the midpalatal suture undergoes a gradual stretching process facilitated by orthodontic appliances. In the second approach, the suture is opened and spaced apart by surgery. It was considered that adolescents and young adults could no longer be treated using the RME method and should be treated with the SARME method [ 2 ].

However, recent findings using the two aforementioned markers (DMPS and SMPS), derived from cone beam computed tomography (CBCT) radiography, have revealed that even patients in adolescence and young adulthood, even up to the age of 26, can have low levels of DMPS and SMPS. Thus, they could be treated using the RME method and do not require invasive procedures [ 3 ].

In research conducted by scientists, CVM, DMPS, and SMPS growth indicators were reviewed separately and comparatively. For example, Ramoglu *et al*. [ 4 ] investigated SMPS in conjunction with the patient's chronological age. Jeon *et al*. [ 5 ] explored the application of DMPS in the treatment of palatal expansion. In a similar vein, Dani *et al*. [ 6 ] investigated the relationship between DMPS and skeletal age. In 2018, Hosni *et al*. [ 7 ] examined the relationship between CVM and skeletal growth velocity. In 2019, Sayar *et al*. [ 8 ] studied SMPS as a factor in the treatment of palatal expansion.

However, no research has been conducted to compare and identify relationships between these three parameters and the patient's chronological age independently in men and women. In the present study, these correlations were obtained in pairs of parameters at different ages and sexes, separately. Besides, the findings of this study could offer clinicians a refined approach to treatment planning for maxillary expansion in both adolescents and young adults. The integration of measurements from DMPS, SMPS, and CVM into a comprehensive assessment model enables orthodontists to more accurately determine the ideal timing and selection of expansion techniques, such as RME versus SARME. This model enables a more customized approach that extends beyond chronological age, potentially reducing the risks of adverse outcomes associated with an improper technique selection. 

## Materials and Method

This study was a cross-sectional study. It was conducted in the Department of Orthodontics, School of Dentistry, Shiraz University of Medical Science, between 2021 and 2022. The study was approved by the Research Ethics Committee of Shiraz University (code: IR. SUMS. DENTAL.REC.1400.101).

A retrospective analysis was conducted on the archived CBCT scans of 80 patients with an age range of 7 to 30 years. For the purpose of comparative analysis, patients were divided into the following age groups: 7-10, 10-13, 13-16, 16-19, 19-22, 22-25, 25-28, and 28-30 years. These age groupings enabled the analysis of growth and developmental variations across the sample, thereby facilitating a more comprehensive understanding of the CVM and DMPS stages at different maturation intervals. All the CBCT scans were evaluated by an undergraduate dental student and an expert radiologist. Two specialists read the existing CBCTs, and the intraclass correlation coefficient (ICC) value was between 0.8-1, indicating a high degree of similarity. Consequently, any observed variations were deemed to be non-significant. The sample size was estimated using the sample size tables for logistic regression, with α=0.05, and a power of 80%. 

In the target population, three parameters of the CVM stage, stage of midpalatal suture morphology, and midpalatal suture density were collected.

**Figure 1:**
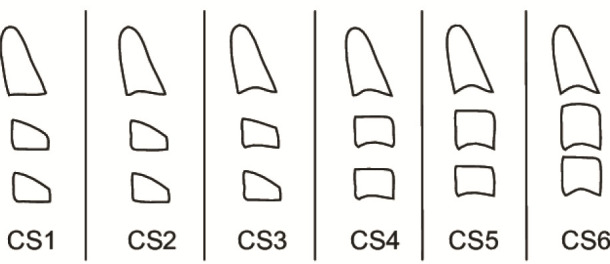
Cervical vertebra maturation) stage (CS) measurement

Based on the morphology of second cervical vertebra (C2), third cervical vertebra (C3), fourth cervical vertebra (C4), and sixth cervical vertebra (C6), maturational stages of the cervical vertebrae could be determined. They were collected from the lateral cephalography of patients (Figure 1). 

**Figure 2:**
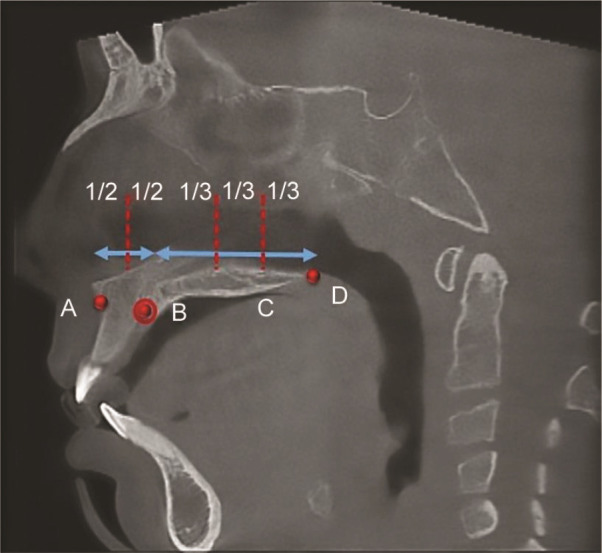
Four points are determined on the sagittal slice. **a:** A Point, the most posterior point at the anterior edge of the maxillary alveolar process. **b:** B Point, center of the incisive foramen. **c:** C Point, the distance between B and D was divided into three thirds and point c was at the junction between the anterior two- thirds and posterior thirds. **d:** D Point, The most posterior point at the posterior edge of the hard palate.

The DMPS were classified using a standard evaluation of Honsfield Unit (HU) values obtained from CBCT scans. These HU values provided a quantitative measure of bone density at different stages. DMPS values were classified based on HU ranges that correlate with varying degrees of suture maturation. It is noteworthy that higher HU values are indicative of advanced bone density and maturity. This classification was based on previously validated criteria in the literature [ 9 ], which correlate HU thresholds with specific stages of suture closure and ossification. In the present study, the DMPS was measured using the Honsfield unit in CBCT viewer software on the sagittal slice passing through the anterior and posterior nasal spine in four points (Figure 2).

**Figure 3:**
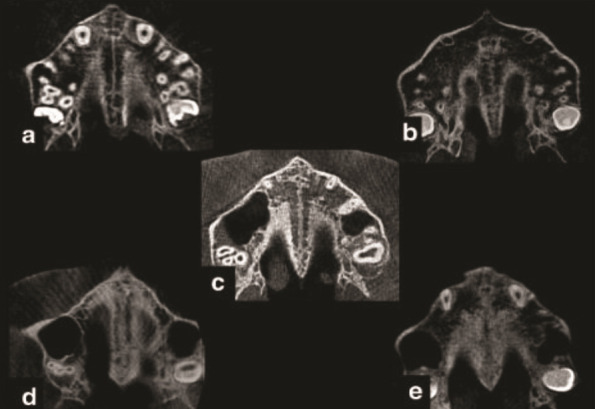
Morphological maturation stages of mid palatal suture. **a:** Stage A, one relatively straight high- density midpalatal suture line. **b:** Stage B, one scalloped, high density line at the midline. **c:** Stage C, two parallel, scalloped high- density line that are closed to each other, separated in some areas by small low- density spaces. **d:** Stage D, two scalloped, high density lines at the midline on the maxillary portion of the palate. **e:** Stage E, sutural fusion, and the midpalatal suture cannot be identified.

To determine the mid-palatal suture (MPS) morphology stages, the most axial central cross-sectional slices from the nasal to the oral surface were used. These were taken from CBCT images and had 5 stages (Figure 3).

Finally, the statistical analysis was performed using the SPSS software (version: 24). Statistical tests including descriptive statistics were used to determine the frequency. The non-parametrical tests such as Pearson and Spearman's rho tests were used to examine the correlation between the study indices. *p* Values less than 0.05 were considered statistically significant. 

## Results

In this study, the archived CBCT scans of 80 patients aged 7 to 30 were evaluated retrospectively.

As indicated in Table 1, an analysis of age-specific frequencies for CVM stages revealed trends across developmental age groups. Notably, the CVM stage 3 (Cs3) exhibited a higher prevalence among younger groups (7–13 years), while older age groups (22–30 years) indicated increased frequencies of Cs5 and Cs6. Specifically, Cs3 peaks in the 10-13 age group (70%), and Cs5 reaches its highest frequency (70%) in the 25-28 group, with minimal to no occurrences of earlier stages (Cs2 and Cs3) in ages 22-30.

**Table 1:** Descriptive statistics of CVM stages and SMPS in different age groups

Age	CVM stage						SMPS stage				
	Cs2	Cs3	Cs4	Cs5	Cs6	Total	B	C	D	E	Total
7-10	3(30%)	5(50%)	2(20%)	0(0)	0(0)	10(100%)	4(40%)	6(60%)	0(0%)	0(0%)	10(100%)
10-13	2(20%)	7(70%)	1(10%)	0(0)	0(0)	10(100%)	4(40%)	6(60%)	0(0%)	0(0%)	10(100%)
13-16	1(10%)	4(40%)	4(40%)	1(10%)	0(0)	10(100%)	0(0%)	7(70%)	3(30%)	0(0%)	10(100%)
16-19	0(0)	1(10%)	5(50%)	2(20%)	2(20%)	10(100%)	0(0%)	6(60%)	4(40%)	0(0%)	10(100%)
19-22	0(0)	3(30%)	6(60%)	1(10%)	0(0)	10(100%)	0(0%)	4(40%)	6(60%)	0(0%)	10(100%)
22-25	0(0)	0(0)	5(50%)	5(50%)	0(0)	10(100%)	0(0%)	3(30%)	7(70%)	0(0%)	10(100%)
25-28	0(0)	0(0)	2(20%)	7(70%)	1(10%)	10(100%)	0(0%)	0(0%)	7(70%)	3(30%)	10(100%)
28-30	0(0)	0(0)	1(10%)	6(60%)	3(30%)	10(100%)	0(0%)	0(0%)	5(50%)	5(50%)	10(100%)

CVM: cervical vertebra maturation; SMPS: stage of maturation of midpalatal suture

In the case of SMPS stages, stage C was predominant among subjects aged 7-13, while stages D and E became more prevalent among subjects aged 19-30.

Notably, stage D reached its peak among subjects aged 25-28 (70%) and exhibited a balanced presence of stages D and E (50% each) among subjects aged 28-30. 

These distributions demonstrated a progressive increase in CVM and SMPS stages with advancing age.

Table 2 provides a synopsis of the DMPS values across various age demographics, illustrating a progressive escalation in density with increasing age. In the subsequent age group of 13-16, a significant increase was observed, with values ranging from 501 to 791 Hu (mean=602, SD=151.5). This trend persists in the oldest group (28-30 years), where the DMPS values reach a maximum range of 797-924 Hu, with a mean of 866.2 and SD of 63.7. Overall, higher age groups exhibit increased mean DMPS values, indicating a significant age-related rise in midpalatal suture density.

**Table 2:** DMPS status in different age groups

Variable	DMPS			
Age	Min	Max	Mean±	SD
7-10	401	513	463.9	56.9
10-13	403	518	465.8	57.7
13-16	501	791	602	151.5
16-19	631	867	756.5	118.2
19-22	658	803	711.4	74.9
22-25	687	851	764.4	82.1
25-28	743	882	809.2	69.5
28-30	797	924	866.2	63.7

DMPS: density of the midpalatal suture

Nonparametric analysis indicated significant correlations among age, DMPS, SMPS, and CVM for both males and females. In females, age demonstrated a strong positive correlation with DMPS (r= 0.791), SMPS (r= 0.667), and CVM (r= 0.750). Similarly, males exhibited significant correlations, with age correlating positively with DMPS (r= 0.832), SMPS (r= 0.693), and CVM (r= 0.795). Additional inter-variable correlations were observed in both groups: DMPS and SMPS were positively correlated (females: r= 0.691; males: r= 0.743), and CVM was significantly correlated with SMPS (females: r= 0.721; males: r= 0.784) (Table 3).

**Table 3:** The correlation between age, DMPS, SMPS, and CVM in males and females

Variables	Female				Male			
	Age	CVM	DMPS	SMPS	Age	CVM	DMPS	SMPS
Age	-	-	-	-	-	-	-	-
CVM	0.750	-	-	-	0.795	-		-
DMPS	0.791	0.769	-	-	0.832	0.805		-
SMPS	0.667	0.721	0.691	-	0.693	0.784	0.743	-

DMPS: density of the midpalatal suture; SMPS: stage of maturation of midpalatal suture; CVM: cervical vertebra maturation

In both female and male groups, DMPS values exhibited an upward trend from CVM2 to CVM6. In females, the highest DMPS value was observed in CVM6 (Max=924), while the lowest was in CVM2 (Min=441),

with mean DMPS values rising progressively across stages. A similar trend was observed in the male group. DMPS peaked in CVM6 (Max= 972) and was lowest in CVM2 (Min= 461). A significant correlation between CVM and DMPS was noted in both sexes (*p*< 0.05). The complete set of data can be found in Table 4.

**Table 4:** The correlation between CVM and DMPS in females and males

CVM	Female				Male			
	DMPS				DMPS			
	Min	Max	Mean±SD	*p* Value	Min	Max	Mean±SD	*p* Value
CVM2	441	491	466±25	<0.05	461	547	504±39.8	<0.05
CVM3	542	687	614.5±74		534	699	616.5±79.9	
CVM4	683	832	757.5±75.3		705	864	784.5±80.2	
CVM5	801	867	834±31		850	883	866.5±16.7	
CVM6	843	924	898.5±76.1		901	972	936.5±35.2	

CVM: cervical vertebra maturation; DMPS: density of the midpalatal suture

Furthermore, a significant correlation (*p*< 0.05) was found between CVM and SMPS in both the female and male populations (Table 5). 

**Table 5:** The relationship between CVM and SMPS in females and males

CVM	Female						Male					
	SMPS						SMPS					
	B	C	D	E	Total	*p* Value	B	C	D	E	Total	*p* Value
CVM2	1	0	0	0	1 (100)	<0.05	4 (80)	1 (20)	0	0	5	<0.05
CVM3	1 (7.5)	(923) 11	0	0	12 (100)		1 (125)	7 (87.5)	0	0	8 (100)	
CVM4	0	6 (40)	9 (60)	0	15 (100)		0	6 (546)	5 (45.4)	0	11 (100)	
CVM5	0	1 (607)	11(73.3)	3 (20)	15 (100)		0	0	6 (85.8)	1 (14.2)	7 (100)	
CVM6	0	1 (25)	1 (25)	2 (50)	4 (100)		0	0	0	2 (100)	2 (100)	

CVM: cervical vertebra maturation; SMPS: stage of maturation of midpalatal suture

A strong correlation was found between SMPS and DMPS in males (*p*< 0.05). Thus, the minimum was related to B and the maximum was related to E (Table 6).

**Table 6:** The relationship between DMPS and SMPS in females and males

SMPS	Females				Males			
	DMPS				DMPS			
	Min	Max	Mean±SD	*p* Value	Min	Max	Mean±SD	*p* Value
B	441	542	491.5±50.5	<0.05	461	534	497.5±51.8	lt;0.05
C	574	843	723.5±49.6		547	809	678±131.6	
D	783	896	839.5±56.4		830	876	863±23.9	
E	851	924	887.5±36.1		883	972	927.5±44.8	

DMPS: Density of the midpalatal suture; SMPS: Stage of maturation of midpalatal suture

## Discussion

Deciding between RME and SARME to expand the narrow maxilla remains a challenge for clinicians, particularly in adolescent and young adult patients [ 10 - 11 ]. Since MPS maturation varies widely among individuals, chronological age alone is insufficient for predicting treatment outcomes. Thus, individualized assessment of MPS is required before treatment initiation [ 9 , 12 ]. 

This study aimed to determine whether changes in DMPS correlate with MPS morphological maturation stages, thereby reinforcing the value of this classification for clinical decision-making. Previous research indicated that DMPS was a primary factor influencing MPS resistance to expansion forces, where higher density indicates greater resistance [ 11 - 13 ]. Grünheid *et al*. [ 14 ] affirmed DMPS as a significant predictor of RME outcomes. However, standardization among CBCT machines is limited, causing variations in HU measure ments between studies [ 15 ]. These inconsistencies complicate the comparison of DMPS values derived from different CBCT machines and limit their use for determining whether RME or SARME is more appropriate.

In this study, all CBCT images were obtained using a single device (Scanora 3D, Botspot Company, Germany) with uniform exposure settings. It was also established that there was a direct and significant relationship between the voxel gray values obtained from the Scanora 3D device and actual HUs from multislice CT [ 16 ]. Furthermore, some exposure protocols of certain devices revealed relatively steady gray values that could be referred to as HU and density [ 16 ]. The MPS maturation stages proposed by Angelieri *et al*. [ 17 ] provided a practical, reproducible classification across different CBCT machines. However, a limitation of this approach is the lack of direct comparability between histological and CBCT-based suture morphologies, necessitating further validation studies [ 12 ]. Angelieri’s classification suggested that RME treatment was effective in stages A, B, and C, while SARME is recommended for stages D and E [ 17 ]. The findings of the present study supported this classification, showing significantly higher DMPS values in stages D and E than in earlier stages.

This study also evaluated the relationships between SMPS, DMPS, and CVM parameters across age groups and between sexes, revealing significant correlations among these measures. In both sexes, the correlation between age and DMPS was the strongest (r= 0.791 in females and r= 0.832 in males), followed by SMPS (r= 0.667 in females and r= 0.693 in males). In 2016, Knaup *et al*. [ 18 ] reported a relationship between MPS and CVM for all populations. However, they did not analyze age-specific differences. Similarly, Grünheid *et al*. [ 19 ] found a strong correlation between CVM and DMPS in both men and women groups, which was in line with the findings of the present study. Dani *et al*. [ 6 ] reported a significant relationship was found between CVM, DMPS, and age for individuals aged 8-18, which was in agreement with our finding.

Besides, all measured correlations between age, CVM, DMPS, and SMPS were slightly stronger in men. Hosni *et al*. [ 7 ] identified a positive correlation between CVM and SMPS; however, Sayar *et al*. [ 8 ] found no significant correlation. Nevertheless, the findings of the present study supported a generally positive correlation among age, CVM, SMPS, and DMPS, by examining the relationships in different age groups and both sexes. 

Although DMPS can be calculated using CBCT, variations in gray density values across different CBCT scanners suggest that absolute DMPS values may not be reliable for fully determining MPS maturation stages. Nonetheless, the positive association between DMPS and MPS morphological maturation could support Angelieri’s suggestion that treatment planning between RME and SARME in adolescents and young adults should consider these classifications when deciding between RME and SARME in adolescents and young adults [ 9 ].

In fact, this study addresses the critical question: “How can orthodontists make evidence-based decisions on maxillary expansion based on individualized bone maturity, rather than relying solely on age?” By highlighting the correlation between DMPS, SMPS, and CVM stages, this study offers a quantitative foundation for determining whether a patient is better suited for RME or SARME. Using these metrics can improve treatment predictability and efficacy, particularly in cases where chronological age alone is insufficient for treatment planning. 

One limitation of this study was the relatively small sample size, with a restricted age range and a specific geographic population. Although the present study provided valuable insights into the correlation of CVM, DMPS, and SMPS across age groups, the findings might not be fully generalizable to other populations with different demographic or genetic backgrounds. Future studies involving larger, more diverse samples could enhance the applicability of these findings and further validate the use of these parameters across broader patient groups.

## Conclusion

This study identified significant relationships between SMPS, DMPS, and CVM across various age groups and between male and female subjects. The findings revealed a highly significant relationship between these four parameters across all ages and in both sexes. The findings of this study indicate that the strongest correlation was observed between age and DMPS, while the weakest correlation was observed between age and SMPS. This suggests that DMPS can be predicted based on the patient's age and vice versa to a certain extent. Although the overall correlation between age and SMPS was strong, SMPS conjecture based on age and vice versa was less probable than in the previous case. Furthermore, the correlations among these parameters were generally stronger in males than in females, indicating a greater likelihood of a relationship in male patients.
